# Inflammation and Endotyping in Chronic Rhinosinusitis—A Paradigm Shift

**DOI:** 10.3390/medicina55040095

**Published:** 2019-04-05

**Authors:** Sinead Ahern, Anders Cervin

**Affiliations:** 1 UQ Centre for Clinical Research, The University of Queensland, Herston, QLD 4029, Australia; a.cervin@uq.edu.au; 2 Faculty of Medicine, The University of Queensland, Herston, QLD 4006, Australia

**Keywords:** chronic rhinosinusitis, CRS, inflammation, endotyping

## Abstract

Chronic rhinosinusitis (CRS) is a heterogeneous chronic inflammatory condition of the paranasal sinuses and nasal passage. It is characterized as inflammation of the sinonasal passage, presenting with two or more symptoms (nasal blockage, secretions, facial pain and headaches) for more than 12 weeks consecutively. The disease is phenotypically differentiated based on the presence of nasal polyps; CRS with nasal polyps (CRSwNP) and CRS without nasal polyps (CRSsNP). Traditionally, CRSwNP has been associated with a type 2 inflammatory profile, while CRSsNP has been associated with a type 1 inflammatory profile. Extensive work in characterizing the inflammatory profiles of CRS patients has challenged this dichotomy, with great variation both between and within populations described. Recent efforts of endotyping CRS based on underlying pathophysiology have further highlighted the heterogeneity of the disease, revealing mixed inflammatory profiles coordinated by a number of inflammatory cell types. This review will highlight the current understanding of inflammation in CRS, and discuss the importance and impact of refining this understanding in the development of appropriate treatment options for CRS sufferers.

## 1. An Introduction to Chronic Rhinosinusitis 

Chronic rhinosinusitis (CRS) is a heterogeneous chronic inflammatory condition of the paranasal sinuses and nasal passage. It is considered one of the most prevalent chronic diseases worldwide, conservatively affecting around 8.5% of the Australian population and placing significant direct and indirect healthcare costs on economies globally [1,2]. CRS is characterized by the presence of at least two of nasal blockages and secretions, facial pain, and headaches for more than 12 weeks [3]. Endoscopic or CT interpretation of the state of sinus disease is used as a diagnostic tool and allows the disease to be phenotypically differentiated into two classes; CRS with nasal polyps (CRSwNP) and CRS without nasal polyps (CRSsNP) [4].

Current treatment protocol includes saline nasal irrigation, antibiotics, and topical and oral corticosteroids. Where pharmacological intervention is insufficient, endoscopic sinus surgery is performed, with the aim of widening the openings of the sinuses, removing inflammatory tissue, reducing inflammatory load, and in CRSwNP, removing nasal polyps [5]. Despite these guidelines, around 30% of CRS patients experience difficulties managing symptoms [3].

## 2. The Role of the Immune System in the Upper Airways

### 2.1. CRS—A Microbiome in Dysbiosis? 

Until recently, healthy human sinuses were considered sterile environments, with CRS developing in response to bacterial infection [3]. A burgeoning focus on the human microbiome, the microorganisms that exist in and on human tissue, has led to a paradigm shift when considering what constitutes “healthy” sinuses. It is now understood that healthy sinuses are comprised of a varied and diverse local bacterial population acting in symbiosis, including low levels of bacteria that have typically been classified as pathogenic [6,7]. A number of studies have aimed to characterize the microbiome of the sinuses in both healthy and CRS patient cohorts. While the sinus microbiome of healthy and CRS affected populations appear heterogeneous and unique to the individual, decreased bacterial diversity, and a noticeable shift in the proportion of respective taxa has been identified in CRS patients [8,9,10]. Commensal taxa that have often been reported as depleted in CRS patients include *Bacteroidetes spp.*, *Prevotella spp.*, *Lactobacillus spp.*, *Peptoniphilus spp.*, *Propionibacterium acnes*, *Acinetobacter johnsonii* and *Corynebacterium confusum*. Taxa found to be enriched include *Pseudomonas spp.*, *Corynebacterium spp.*, *Streptococcus spp.*, *Staphylococcus aureus* (*S. aureus*), *Propionibacterium acnes* and *Haemophilus influenzae (H. influenza)* [6,7,8,9,10,11,12,13]. Differences in microbiome within the CRS population are also important to consider, with nasal polyps providing niche microenvironments for bacterial colonization. Notably, CRSwNP is associated with increased *S. aureus* presence, in comparison to CRSsNP [10,14,15,16].

Increased richness of ‘pathogenic’ bacteria and a loss of protective bacterial strains may be a driving feature of the local immune response seen in CRS. Interestingly, bacterial species, such as *S. aureus*, have been suggested to play a protective role in the sinus microbiome under normal conditions; however, in a state of dybsiosis, they are associated with an increased local immune response and disease severity [17]. Thus, loss of a balanced and diverse sinus microbiome seems to be a significant player in CRS; however, whether this dysbiosis is a causative or propagative mechanism remains a point of debate. A state of dysbiosis may lend itself to induction of an inflammatory response, while inflammation itself can create an environment conducive of shifts in the local bacterial population. A more in-depth understanding of host-microbiome interactions, including investigation into the effects of microbial metabolites on host immunity [18], may allow for increased understanding of the CRS inflammatory response.

### 2.2. The Role of the Mucociliary System

The airways are lined with anti-microbial mucus comprised of mucins produced by goblet cells and submucosal glands [19]. A number of microorganisms can be bound by mucins, trapping them in this mucus layer. Coordinated and directional beating of cilia allows the mucus (and the matter it has ‘caught’) to be ‘swept’ from the sinonasal cavity to the oropharynx for clearance, in a process known as mucociliary clearance (MCC) [20,21]. Several pathogenic bacterial taxa are known to produce products that impair ciliary action, reducing capacity for MCC, and increasing bacterial capacity for colonization. *H. influenzae*, *S. aureus* and *Pseudomonas aeruginosa* (*P. aeruginosa*) are commonly enriched in CRS and are known to produce products that interfere with ciliary action [22,23,24]. Furthermore, a build-up of mucus may induce local hypoxia, leading to mucostasis and production of reactive oxygen species, inducing further inflammation in CRS [25].

### 2.3. Innate Immunity and Epithelial Immunity

The upper-airways have a number of protective mechanisms against pathogens and irritants, which are seemingly overcome in CRS. The upper respiratory tract is lined by epithelial cells which utilize tight junctions and adherens junctions to protect underlying immune-reactive tissue from pathogens and irritants [26]. Commensal bacterial species have been associated with reinforcement of epithelial tight junctions and adherens junctions, and production of anti-inflammatory cytokines [11]. A loss or reduction in richness of these commensal species may lead to a reduction in epithelial integrity in CRS patients. Some bacterial species associated with CRS have been shown to directly impact tight junction proteins, reducing epithelial integrity, and allowing increased pathogen detection by local immune mediators [27,28]. Interferon gamma (IFN-γ) and interleukin 4 (IL-4) have been shown to influence epithelial integrity in CRS by interfering with expression of epithelial tight junction proteins [29]. Release of these immune mediators leads to a reduction in epithelial integrity, allowing for increased immune stimulation of sub-epithelial layers, thus creating an inflammatory cycle congruent with the exaggerated response seen in CRS.

### 2.4. Recognition of Non-Self

Where the protective processes of the upper respiratory system fail, or are compromised, microbes persist, and respiratory epithelial cells produce cytokines and chemokines that recruit immune cells and activate inflammatory pathways [25]. Pathogens or foreign substances can also be recognized by toll-like receptors (TLRs) via structures known as pathogen-associated molecular patterns (PAMPS). PAMPS can be a number of different structures including DNA, RNA, chemical products or physical structures that are foreign to the local immune system. Binding of PAMPs to the ligand-binding domain of TLRs leads to downstream signal transduction that stimulates the production of inflammatory cytokines and chemokines. These factors promote antigen presentation, induction of co-stimulatory molecules of dendritic cells, and recruitment of immune cells [30].

## 3. CRS—A Chronic Inflammatory Disease

### 3.1. The Role of T-Effector Cells

A number of T effector cells play an important role in modulating the immune response of the upper airways, with T helper 1 (Th1), T helper 2 (Th2), T helper 17 (Th17), T helper 22 (Th22) and T regulatory (Treg) cells predominating in CRS [31]. Th1 cells mature in response to an IFN-γ and IL-12 environment, and produce IFN-γ and IL-2 as part of a type 1 inflammatory response. Th2 cells maturation is induced in an IL-4 environment, and the subsequent type 2 inflammatory response is characterized by the production of inflammatory cytokines IL-4, IL-5 and IL-13 [32]. A type 3 response is mediated by Th17 cells, which mature in response to Transforming Growth Factor β (TGF-β) and IL-6. This response is characterized by the production of IL-17 and IL-22. Th22 cells mature in response to an IL-6 environment, and produce IL-22 [33]. Treg maturation is stimulated by TGF-β and IL-2, and leads to production of TGF-β [34].

### 3.2. The Geographical Conundrum 

Until recently, CRSwNP was thought to be characterized by type 2 inflammation, while CRSsNP was thought to be characterized by type 1 inflammation. Studies profiling inflammatory mediators in CRS patients have found significant differences in inflammatory cytokine expression, initially between geographical centers, and now within classical CRS phenotypes. CRSwNP is strongly skewed towards a type 2 response in American and European patient cohorts; however, this relationship is not mimicked in Asian populations. Rather, Asian CRSwNP populations, with the majority of the data coming out of China, tend towards neutrophilic inflammation. Similarly, type 1 inflammation, measured by IFN-γ expression, used to define CRSsNP. IFN-γ has been reported to be elevated in Belgian, Chinese and Korean CRSsNP populations [4,35,36,37], while studies in Japan, China and America reported no significant elevation of this marker [38]. Tan et al. [39] reported no significant difference in IFN-γ levels in a controlled study comparing only ethmoid tissue of healthy, CRSsNP and CRSwNP patients, unlike previous work in their lab [40] and other published data [35,36] which compared IFN-γ levels between healthy, CRSsNP and CRSwNP patient cohorts, each with different tissue sample sites. While Chinese cohort studies have varied reports of IFN-γ elevation, a strong neutrophilic dominance, regardless of phenotype, has been highlighted in Chinese patients [31,37,39,41].

### 3.3. Type 2 Inflammation Is Well Characterized

**Figure 1:**
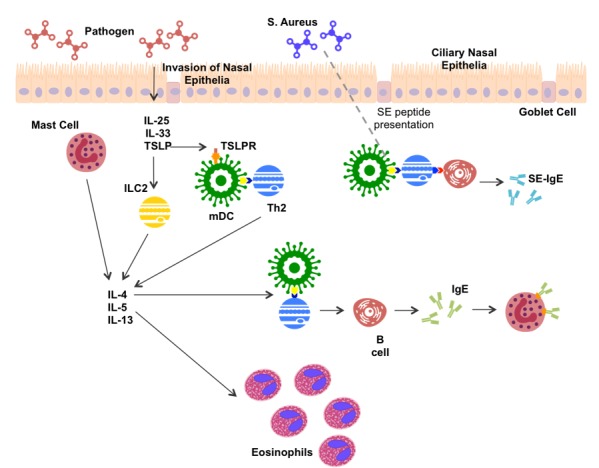
Potential mechanism of type 2 inflammation in CRS patients. *S. aureus, Staphylococcus aureus*; IL-, Interleukin; TSLP, Thymic Stromal Lymphopoietin; TSLPR, TSLP Receptor; Th2, T helper 2 cell; ILC2, Innate-like cell 2; IgE, Immunoglobulin E; SE, *S. aureus* enterotoxin; mDC, Myeloid Dendritic cell.

In CRS, the type 2 inflammatory response is fairly well characterized (Figure 1). Recognition of foreign matter stimulates nasal epithelial cells to secrete thymic stromal lymphopoietin (TSLP), IL-25 and IL-33 [42]. TSLP, IL25 and IL-33 stimulate secretion of IL-4, IL-5 and IL-13 from epithelial and mucosal mast cells [20,43,44]. TSLP and IL-33 can further induce type 2 cytokine production in innate lymphoid cells (ILC2s) [45]. TSLP has been suggested to stimulate myeloid dendritic cells (mDCs) by binding to the TSLPR on the mDC membrane [44,46]. Once activated, mDCs are able to present antigen and co-stimulatory signals to induce CD4+ T cell differentiation. Mast cell and ILC2 production of IL-4 directs the differentiation of CD4+ T cells toward Th2 production [44]. Th2 cells produce type 2 cytokines as the hallmark feature of the type 2 inflammatory response. Thus, a strong IL-4 environment is created, promoting further Th2 expansion.

IL-5 plays a major role in eosinophil infiltration, leading to production of eosinophilic extracellular traps, inflammatory products, and toxic proteins [47]. IL-4 and IL-13 have been associated with increased production of the genes *MUC5AC* and *MUC5B*, which encode for the production of mucins [48], and pendrin, an epithelial anion transporter, which results in increased mucus production [49]. Increased mucus production can amplify local inflammation by inducing hypoxia [50], as well as being a significant contributor to CRS symptomology.

Th2 cells present antigen and co-stimulatory signals to B cells aiding in antibody production, while IL-4 produced by Th2 cells, mDCs and ILC2s induces antibody isotype switching to Immunoglobulin E (IgE) [34]. *S. aureus*, a commonly up-regulated bacteria in CRS (predominantly CRSwNP), has been shown to bind TLR-2 leading to an increase in type 2 cytokine production [51]. Further, production of *S. aureus* enterotoxin (SE) amplifies the type 2 response, acting as a super antigen, and leading to SE-IgE production [16].

### 3.4. Non-Type 2 Inflammation—A New Concept

**Figure 2:**
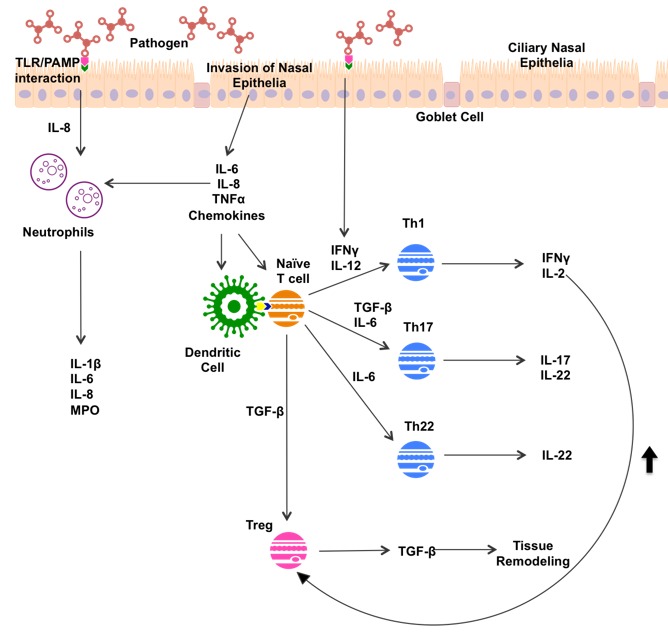
Potential mechanisms of non-type 2 inflammation in CRS patients. TLR, Toll-like Receptor; PAMP, Pathogen Associated Molecule; IFN-γ, Interferon gamma; IL-, Interleukin; MPO, Myeloperoxidase; TNFα, Tumor Necrosis Factor alpha; Th1, T helper 1 cell; Th17 T helper 17 cell; Th22, T helper 22 cell; Treg, regulatory T cell; TGF-β Transforming Growth Factor.

Non-type 2 inflammation in CRS displays a mix of mainly type 1 and type 3 inflammation, often associated with significant neutrophil infiltration (Figure 2). Pathogen invasion of nasal epithelia leads to release of IL-6, IL-8, Tumor Necrosis Factor α (TNFα), and various chemokines by nasal epithelia. PAMP/TLR interactions have been shown to simulate IFN-γ and IL-8 production [30]. These innate immune responses recruit immune cells to the sinuses, and sway the subsequent immune response.

Both PAMP/TLR interactions and nasal epithelial cells secrete IL-8, which recruits neutrophils to the area [35]. Neutrophils release a variety of products, including inflammatory cytokines IL-1β, IL-6 and IL-8, and myeloperoxidase (MPO), an enzyme released by neutrophil granulocytes [52]. IFN-γ, secreted by epithelial cells in response to pathogen recognition, directs CD4+ T cell differentiation toward Th1 maturation [30]. Th1 cells mediate the type 1 inflammatory response through production of IFN-γ and IL-2. Epithelial secretion of IL-6 directs CD4+ T cell differentiation towards Th17 and Th22 production. Th17 cells go on to secrete IL-17 and IL-22, while Th22 cells secrete IL-22 alone [33]. IL-22 is known to stimulate production of antimicrobial peptides and mucin 1 in an inflammatory environment [53]. In response to different markers, increased mucus production is seen in type 2 and non-type 2 inflammation; however, induction of hypoxic microenvironments can perpetuate inflammatory processes in both responses [50].

Up-regulation of Tregs has been noted in CRSsNP in comparison to healthy patients, and a down-regulation of Tregs in CRSwNP [36]. Further, Tregs are typically up-regulated in a type 1 environment, with Th1-produced IL-2 vital in Treg maturation [54]. Tregs play a vital role in immune regulation, down-regulating Th1 and Th2 function, and producing the anti-inflammatory cytokine IL-10 [54]. Tregs produce TGF-β, a member of the transforming growth factor cytokine superfamily, which has been suggested to play a key role in tissue remodeling in CRSsNP [55]. TGF-β is involved in induction and proliferation of fibroblasts, and the upregulation of extra cellular matrix synthesis [56], contributing to remodeling of airway epithelia that can cause symptomatic burden in CRSsNP sufferers [55]. TGF-β promotes differentiation of CD4+ T cells toward Th17 and Treg maturation [36,57]. The importance of the role of Tregs in CRS is still in question, given its capacity to reduce inflammation by IL-10 production, but also to contribute to airway remodeling and fibrosis as a result of TGF–β production.

The already complex heterogeneous disease state of CRS is further complicated by presence of allergic and fungal rhinitis, cystic fibrosis and, the most commonly reported co-morbidity, asthma. Each of these disease states harbors their own unique immune response, and thus contribute to increasingly specific immunological profiles in patients, making distinct characterization of CRS pathophysiology difficult.

## 4. The Emergence of Endotyping

In recent years, there has been a strong focus on the characterization of the immune response in CRS patients, known as endotyping. This approach to disease classification is paving the way for unique treatment options that are based on underlying pathophysiology, rather than traditional phenotypic classification. It has been instrumental in pushing past the traditional CRSwNP/Type 2 and CRSsNP/Type 1 dichotomy, and has highlighted the inflammatory heterogeneity of the disease.

### 4.1. Endotyping by Inflammatory Markers

Endotyping performed by Tomassen et al. [58], based on biomarker cluster analysis of inflammatory markers, highlighted a broad distinction between type 2 and non-type 2 CRS cytokine profiles. A total of 10 unique clusters were identified from the study, with six clusters displaying markers typical of type 2 inflammation, and four clusters displaying non-Type 2 associated markers, with IL-5 levels the key determinant of this distinction. Three clusters with high IL-5 levels were identified, two of which were positive for SE-IgE. Further, three sub-types of the non-type 2, or IL-5 negative, endotype were delineated as follows:

1. Neutrophilic inflammation characterized by pro-inflammatory cytokines IL-1β, IL-6, IL-8 and Myeloperoxidase
2. Th17- or Th22- driven inflammation characterized by IL-17, IL-22
3. Th1-driven inflammation characterized by IFN-γ

### 4.2. Endotyping by Clinical Features

Bachert et al. [59,60] considered the findings of Tomassen et al. [58] and highlighted the clinical relevance of comorbidities, and clinical features to the endotyping process. They classified CRS into three endotypes: Non-type 2 inflammation, correlating with the CRSsNP phenotype, low asthma risk, and low recurrence risk; moderate type 2, containing a mix of CRSsNP and CRSwNP, moderate asthma, and recurrence risk; and severe type 2, correlating with the CRSwNP phenotype and high risk of asthma, and disease recurrence. Consideration of co-morbidities provides a useful tool for disease conceptualization, given shared inflammatory mechanisms between co-morbidities and CRS, and the potential immunomodulation of CRS by other inflammatory processes [61].

One of the largest CRS endotyping efforts to date was performed in a Chinese population by Liao et al., who performed cluster analysis on 246 patients, based on 28 clinical variables, and 39 mucosal and molecular variables [62]. Basic endotyping of inflammatory markers was furthered in this study by the inclusion of co-morbidities, and classification of cases based on responsiveness to treatment. The inclusion of these variables in cluster analysis allows stratification of disease severity and pathophysiology, and is a useful tool for future endotyping efforts. Previous work by this group includes profiling inflammation in a Chinese CRS patient cohort, which highlighted geographical differences in cytokine expression, particularly in comparison to American and European cohorts [35,36,37,38,39,41,55,63,64]. A total of seven clusters were identified, including a unique cluster characterized by high levels of anti-inflammatory cytokine IL-10, and a lack of cases that were difficult to treat.

Soler et al. published two papers outlining CRS endotyping based on cluster analysis of clinical makers alone [65,66]. Classical biomarker-based endotyping aims to identify patient inflammatory clusters in order to allow for more targeted treatment selections. The aim of the work by Soler et al. is similarly geared toward treatment selection and response. Cluster analysis based on SNOT-22 score, age and productivity loss identified five patient clusters, three of which responded better to surgical intervention compared to pharmacological intervention. While these findings are interesting, clinical translation of these findings on their own seems unlikely. Despite this, the relevance of clinical markers to endotyping should not be understated, and disease severity and impact on quality of life should be considered. 

### 4.3. Endotyping by Microbial Composition

Association of inflammatory endotypes with microbial composition has also been attempted, with Cope et al. identifying four clusters based on microbial composition, and linking these clusters to inflammatory markers observed within the cluster [67]. Recently published work by Hoggard et al. aimed to delineate inflammatory endotypes, and their associations with microbial compositions in CRS patients [68]. Cluster analysis of inflammatory markers, immune cells, polyp status, and asthma co-morbidity revealed eight distinct clusters, while associations with various microbial changes were identified. The analyses performed in this study not only further challenged the traditional Th1 vs. Th2 dichotomy, but also suggested that a number of key inflammatory markers thought to “characterize” inflammatory endotypes (IL-5, neutrophils, eosinophils) are not necessarily altered in all patients. While the general endotypes delineated reflected those of Tomassen et al. [58], distinction between endotypes on the basis of the type-2 cytokine IL-5 were not made in this study. The results thus suggested a semantic change from “characterizing” or “defining” inflammatory markers, to markers which have “increased incidence” in certain endotypes.

### 4.4. Endotyping by Nasal Secretions

Turner et al. [69] performed the first cluster analysis based on nasal secretions rather than nasal biopsy, highlighting the opportunity for non-invasive endotyping of patients. Analysis of nasal secretions/mucus is not only non-invasive, cheap and easily accessible, but allows for standardization of sample collection which could aid larger multi-center endotyping efforts, which would ultimately allow for effective characterization of CRS endotypes. A fault highlighted by the study team was that all patients in the study cohort had previously undergone endoscopic nasal surgery; however, this limitation is applicable to all endotyping efforts to date. The sampling method used, however, allows for this limitation to be overcome.

### 4.5. Endotyping-Still Under Development

Further, the diversity in endotyping approaches taken by different research groups makes meaningful comparison difficult. There has been significant variability in sampling sites, markers analyzed, analysis methods and statistical methods used. Each factor introduces an additional layer of variability, making it exceptionally difficult to compare endotyping efforts, and to form a genuine idea of CRS pathophysiology as a whole. Ultimately, clinically relevant endotypes cannot be distinguished without significant data. A controlled, uniform multi-site study of CRS pathophysiology would allow for comparable data to be collected, creating a large dataset to accurately cluster patient profiles into meaningful groups.

## 5. Treatment

Endotyping of patients allows for the selection of treatments specific to individual disease state, rather than blanket treatment approaches which may have no positive impact. Bachert et al. highlighted the possibility of endotype driven care in CRS patients, with clinical trials of monoclonal antibodies (mAbs) targeting type 2 inflammatory processes in CRSwNP patients well underway [60]. mAbs targeting type 2 inflammatory profiles predominate biologic therapy, proving more effective than corticosteroid therapy when used appropriately [60]. Omalizumab is an anti-Ig-E mAb which binds IgE, blocking the IgE inflammatory cascade, and has been shown to be reduce nasal polyp score and symptoms in patients with nasal polyps with asthma [70]. Mepolizumab is an anti-IL-5 mAb that binds IL-5, preventing it binding to its receptor. It has been shown to significantly reduce nasal polyp score, and the need for surgical intervention [71,72]. Benralizumab is an anti-IL-5 mAb which binds to the alpha chain of the IL-5 receptor, preventing IL-5 binding and reducing eosinophilia as a result. It is currently undergoing a phase II clinical trial in eosinophilic rhinosinusitis [60]. Dupilumab is an anti-IL-4/IL-13 mAb, which binds the alpha chain of the Interleukin 4 receptor alpha (IL-4Ra), preventing binding of both IL-4 and IL-13. Dupilumab has been shown to improve nasal polyp burden in CRSwNP patients [73].

Comparatively, treatment options for non-type 2 inflammation are lacking. While this cohort is more responsive to macrolide therapy, resistance to antibiotic treatment is becoming increasingly common. Reduced response to corticosteroid treatment is also observed in these patients, and thus there is a distinct need for effective treatments targeting mediators of non-type 2/neutrophilic inflammation. There are currently anti-IL-17 biologics approved for psoriasis treatment, which could be repurposed in appropriate CRS individuals [74], while anti-IL-1 mAbs could also be a potential treatment option for patients with neutrophilic inflammation [75,76].

Current phenotype-based treatment options for CRS leave 30% of patients with unresolved symptoms [3], highlighting the need for targeted options for those unresponsive to standard therapy. There is a promising future for personalized medicine where underlying pathophysiology is determined, and treatment is recommended on the basis of individual inflammatory profiles. Rapid diagnostic tests for sinonasal inflammation could thus be an invaluable tool in the future of endotype-based treatment in CRs patients. Nasal absorption devices, for example, could allow for quick, non-invasive sampling of nasal secretions, which could then undergo inflammatory biomarker analysis. Results of such tests could then be used to determine appropriate treatment options for CRS patients.

## 6. Conclusions

The inflammatory state of CRS is highly heterogeneous, with mixed profiles of type 1, 2 and 3 inflammation seen within classical CRSsNP and CRSwNP phenotypes. Endotyping of CRS disease state is emerging as a useful tool in identifying key inflammatory profiles amongst CRS patients, and provides a unique opportunity for targeted treatment options. A shift in approach to CRS from phenotype to endotype is needed if the burden of CRS on the individual, and on healthcare systems globally, is to be addressed. Diagnostic tools to identify patient inflammatory profiles in a clinical setting would allow for precise and targeted treatment options. Identification of patient inflammatory profiles would allow for selection of targeted therapies, with biological therapies currently being assessed for CRS patients. In order to optimize patient outcomes, further work is needed to understand the inflammatory mechanisms at play in CRS and a global shift in the approach to patient diagnosis away from blanket phenotype distinctions must be taken.
